# Treatment of congenital toxoplasmosis with pyrimethamine combined with sulfadiazine in a Chinese neonate: a case report

**DOI:** 10.3389/fped.2026.1844553

**Published:** 2026-06-26

**Authors:** Ya Hu, Jingsong Wang, Zhenlong Wang, Yu Zhang, XiaoYa He, Min Zhao, Sirao Lu, Bo Zhang, Ya Yang

**Affiliations:** 1 Department of Neonatology, Children’s Hospital of Chongqing Medical University, National Clinical Research Center for Children and Adolescents’ Health and Diseases, Ministry of Education Key Laboratory of Child Development and Disorders, Chongqing Key Laboratory of Child Rare Diseases in Infection and Immunity, Chongqing, China; 2 Department of Pharmacy, Guangyuan Central Hospital, Guangyuan, Sichuan, China; 3 Department of Pharmacy, Children’s Hospital of Chongqing Medical University, National Clinical Research Center for Children and Adolescents’ Health and Diseases, Ministry of Education Key Laboratory of Child Development and Disorders, Chongqing Key Laboratory of Child Rare Diseases in Infection and Immunity, Chongqing, China; 4 Department of Infection, Children’s Hospital of Chongqing Medical University, National Clinical Research Center for Children and Adolescents’ Health and Diseases, Ministry of Education Key Laboratory of Child Development and Disorders, Chongqing Key Laboratory of Child Rare Diseases in Infection and Immunity, Chongqing, China; 5 Department of Ophthalmology, Children’s Hospital of Chongqing Medical University, National Clinical Research Center for Children and Adolescents’ Health and Diseases, Ministry of Education Key Laboratory of Child Development and Disorders, Chongqing Key Laboratory of Child Rare Diseases in Infection and Immunity, Chongqing, China; 6 Department of Radiology, Children’s Hospital of Chongqing Medical University, National Clinical Research Center for Children and Adolescents’ Health and Diseases, Ministry of Education Key Laboratory of Child Development and Disorders, Chongqing Key Laboratory of Child Rare Diseases in Infection and Immunity, Chongqing, China

**Keywords:** case report, congenital toxoplasmosis, follow-up, pyrimethamine, sulfadiazine, treatment

## Abstract

This work reports a case of neonatal congenital toxoplasmosis in which the standard regimen of pyrimethamine combined with sulfadiazine was administered under conditions of limited drug accessibility in China, with multidisciplinary collaboration and long-term follow-up. The infant was a male born at 36⁺⁶ weeks of gestation. Postnatal serological screening and cerebrospinal fluid (CSF) next-generation sequencing (NGS) confirmed the diagnosis of mildly symptomatic congenital toxoplasmosis. In the initial phase of treatment, due to the lack of routine access to standard therapeutic drugs in China, azithromycin was administered as empirical therapy. Subsequently, through multidisciplinary discussions and comprehensive communication with the family, and after obtaining informed consent from the parents who sourced the medications themselves, standard combined therapy with pyrimethamine, sulfadiazine, and folinic acid was initiated, strictly following an individualized regimen. The infant tolerated the treatment well throughout the course, with no significant adverse reactions. Outpatient follow-up at one year showed no adverse events, with normal growth and development assessments. This case demonstrates that, by adopting a standardized approach to address the challenge of drug accessibility, the implementation of this standard treatment regimen is feasible in China, providing a valuable reference for the clinical management of similar rare diseases.

## 1 Introduction

Congenital toxoplasmosis represents a significant intrauterine infectious disease worldwide and continues to pose an important public health challenge in the field of perinatology (1). This infection, transmitted transplacentally by *Toxoplasma gondii (T. gondii)*, threatens the health of fetuses and newborns (2). Maternal infection during pregnancy may result in miscarriage, severe birth defects, or lead to infection-related complications in fetuses, newborns, and young infants with congenital toxoplasmosis, particularly retinal disease that can persist into adulthood (3). Currently, the standard treatment regimen for congenital toxoplasmosis is the classic combination of pyrimethamine and sulfadiazine, supplemented with folinic acid. Due to its well-defined inhibitory effect on the pathogen and favorable tissue penetration, this combination is often recommended as the first-line option (4).

However, the clinical manifestations of congenital toxoplasmosis are diverse and nonspecific (5). The classic triad of chorioretinitis, hydrocephalus, and intracranial calcifications occurs in fewer than 10% of cases (5), which may contribute to diagnostic delay. A more significant challenge is that pyrimethamine is not marketed in China, resulting in a scarcity of clinical experience with the standard regimen for congenital toxoplasmosis. Clinicians are therefore often compelled to resort to alternative agents such as macrolides, including azithromycin (6). Nevertheless, the evidence supporting azithromycin for congenital toxoplasmosis is limited to a small number of case reports (7), and its efficacy awaits validation in higher-quality studies. Although the combination of pyrimethamine and sulfadiazine remains the guideline-recommended standard therapy (4), its clinical application in Chinese neonates is exceedingly rare owing to limited drug accessibility, and systematic follow-up data are lacking. Here we report a case in which this standard regimen was applied through a standardized clinical pathway under conditions of drug inaccessibility, with completion of long-term follow-up. Our objective is to provide a real-world reference for the management of similar rare diseases in China.

This manuscript reports a case of congenital toxoplasmosis presenting primarily with jaundice involving the face, neck, and trunk upon admission, with no fever, cough, or dyspnea during the course of illness. In this case, the infant was initially treated with azithromycin alone. Subsequently, after excluding contraindications, therapy was transitioned to pyrimethamine-sulfadiazine, followed by monitoring for adverse reactions and comprehensive follow-up, ultimately resulting in a favorable outcome. This work aims to accumulate real-world experience regarding the use of the pyrimethamine-sulfadiazine regimen in the face of drug accessibility challenges in China, and to offer guidance for the treatment of congenital toxoplasmosis in the domestic setting.

## 2 Case presentation

The infant was a male born at 36⁺⁶ weeks of gestation, admitted because “serological screening suggested positive *Toxoplasma* antibodies”. The infant was delivered by cesarean section at an external hospital 45 h prior to admission due to severe intrahepatic cholestasis of pregnancy. The birth weight was 3,280 g. The Apgar scores, as well as the characteristics and volume of amniotic fluid, were unknown. The umbilical cord and placenta were normal, and there was no history of intrauterine distress or premature rupture of membranes. In the early morning of the day of admission, testing at the birth hospital revealed positive *Toxoplasma* antibodies on the TORCH panel (specifically, *Toxoplasma* IgM antibody at 34.7 AU/mL and IgG antibody at 400.0 IU/mL). No specific treatment was administered prior to admission. Since the onset of illness, the infant had no fever, abdominal distension, diarrhea, or bloody stools, with no tachypnea, dyspnea, or cyanosis, and no cough, frothing, seizures, or convulsions.

The infant’s mother had blood type B, RhD positive. She had a pre-pregnancy history of hypothyroidism and had been receiving long-term oral levothyroxine sodium therapy, with regular monitoring of thyroid function during pregnancy that she reported to be within the normal range. She had one previous history of pregnancy loss. The family kept a cat at home. During this pregnancy, the mother experienced episodes of hand and foot cramping and received supplementation with vitamin D and calcium. She was diagnosed with gestational diabetes mellitus, which was managed by dietary control. Additionally, she was diagnosed with intrahepatic cholestasis of pregnancy and received treatment with ursodeoxycholic acid, polyene phosphatidylcholine, and ademetionine 1,4-butanedisulfonate. There was no history of hypertension, anemia, hepatitis, AIDS, syphilis, tuberculosis, or other conditions during pregnancy, nor was there any history of blood transfusion or hormone use. Regarding prenatal examinations, no non-invasive prenatal testing or amniocentesis was performed during pregnancy. Infectious disease screening (for hepatitis, AIDS, and syphilis) yielded negative results.

Upon admission, physical examination revealed the following: temperature 37.3 ℃, respiratory rate 48 breaths/min, heart rate 134 beats/min, blood pressure 79/40 mmHg, and weight 3,090 g. The infant was alert, with appearance and nutritional status consistent with a mature newborn. Jaundice was observed in the skin of the entire body and in the sclera. Patchy purplish red ecchymosis was visible on the left side of the face and below the left lower eyelid. The capillary refill time was 2.5 s. There was no perioral cyanosis. Chest examination revealed slightly coarse breath sounds bilaterally, which were symmetric, with no dry or moist rales audible. Cardiac examination revealed strong heart sounds with a regular rhythm, and a grade II/VI systolic blowing murmur was audible at the left sternal border. Abdominal examination revealed a soft, flat abdomen with no tenderness and no palpable masses. The liver was palpable 1 cm below the right costal margin and 0.5 cm below the xiphoid process, with a soft texture and sharp edge. The spleen was not palpable. There was no shifting dullness, and bowel sounds were normal at approximately 4 times/min. Neurological examination revealed that the rooting reflex, sucking reflex, and grasping reflex were all elicited normally, while the Moro reflex was diminished.

After admission, the infant received supportive care including vitamin K1 to prevent bleeding, phototherapy, and fluid supplementation, along with monitoring of bilirubin levels. Laboratory tests on admission revealed a white blood cell (WBC) count of 12.13 × 10⁹/L (4.3-14.2), C-reactive protein (CRP)＜0.80 mg/L (0–10), and procalcitonin (PCT) of 0.61 ng/mL, total bilirubin was 164.5 μmol/L, predominantly unconjugated bilirubin (158.9 μmol/L). Chest x-ray indicated pneumonia. The neonate’s blood type was B, RhD positive.

On the second day of hospitalization, serological test results returned: cytomegalovirus IgG antibody was 188.6 AU/mL, and *Toxoplasma* IgM antibody was 2.84 S/CO. Echocardiography revealed patent ductus arteriosus and patent foramen ovale. Following consultation with the infectious diseases department, further evaluations were planned to assess potential target organ damage due to *Toxoplasma* infection, including cranial magnetic resonance imaging, ophthalmologic examination, auditory evoked potentials, and cerebrospinal fluid (CSF) analysis [including routine examination, biochemical analysis, and next-generation sequencing (NGS)], and oral azithromycin at 30 mg (10 mg/kg) once daily was initiated.

On hospital day 3, cranial magnetic resonance imaging revealed a persistent cavum septum pellucidum without definite abnormal signal foci within the brain (Figure 1). On the fourth day, ophthalmologic examination revealed a hemorrhagic spot in the left temporal region, with no retinopathy (Figure 2A). CSF routine examination showed no abnormalities, but biochemical analysis revealed an elevated protein concentration (1.52 g/L). Consequently, the dose of azithromycin was reduced to 15 mg (5 mg/kg) once daily orally. On the sixth day, the CSF NGS result returned, indicating *T. gondii* infection, leading to a confirmed diagnosis of congenital toxoplasmosis.

**Figure 1:**
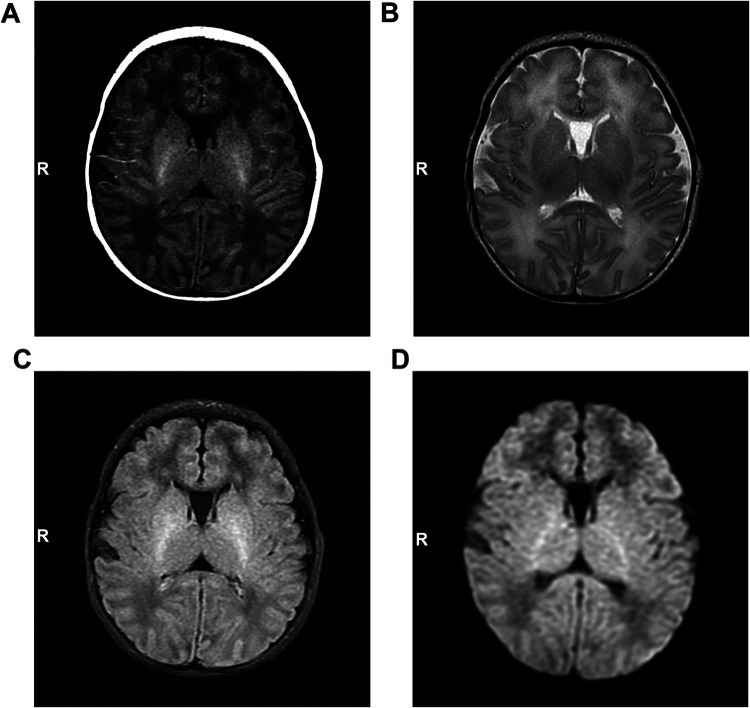
Cranial magnetic resonance imaging (MRI) during hospitalization revealed a persistent cavum septum pellucidum, with no definite abnormal signal foci identified within the brain. No abnormal signal was observed on T1-weighted imaging **(A)**, T2-weighted imaging **(B)**, fluid-attenuated inversion recovery **(C)**, or diffusion-weighted imaging **(D)**.

**Figure 2:**
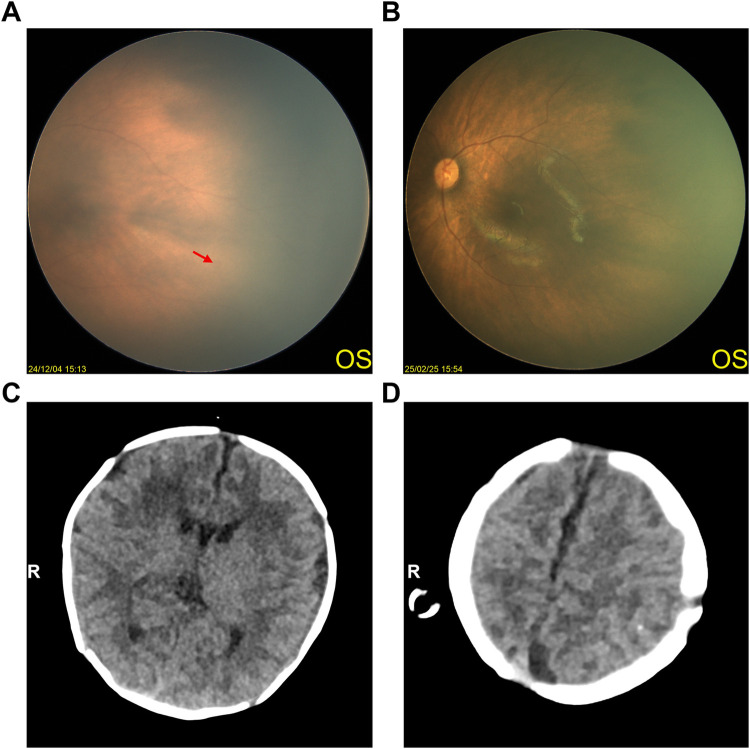
Ophthalmologic and CT examination images. **(A)** Ophthalmologic examination during hospitalization; **(B)** Ophthalmologic examination during follow-up; **(C)** and **(D)** A punctate hyperdense focus in the left parietal lobe, suggestive of a possible microcalcification.

Due to the lack of standard therapeutic drugs for congenital toxoplasmosis in China, namely pyrimethamine and sulfadiazine, along with the absence of established purchasing channels, this regimen represented off-label use and required informed consent from the family. After thorough discussion, the family initially declined to adjust the treatment strategy, primarily citing concerns about the unavailability of standard medications through routine channels in China and the safety of off-label drug use. They requested to continue azithromycin therapy. Accordingly, azithromycin was switched to intravenous administration at 10 mg/kg once daily on the same day, and glucose-6-phosphate dehydrogenase (G6PD) activity and genetic testing were performed in preparation for the potential subsequent use of sulfonamides.

After three days of intravenous azithromycin, further communication was held with the family. We emphasized that, despite the lack of clear clinical symptoms in the infant, based on both domestic and international evidence-based medicine and multidisciplinary consultation recommendations, the standard treatment regimen of pyrimethamine plus sulfadiazine with folinic acid (or trimethoprim-sulfamethoxazole) was still recommended for newborns with confirmed congenital toxoplasmosis to prevent potential long-term neurological sequelae. Concurrently, we explained in detail to the family the necessity of standard treatment, the potential risks of the medications, and the potential consequences of non-intervention. After being informed in detail of the evidence supporting the standard regimen and its potential risks, the family expressed understanding and decided to obtain the drugs through their own means. In subsequent feedback, they indicated that thorough information sharing and involvement in decision-making had progressively built their confidence in the treatment. The following day, repeat CSF NGS for *Toxoplasma* had turned negative, and cranial computed tomography (CT) revealed a small calcification focus in the left parietal lobe (Figures 2C,D). Therefore, azithromycin was discontinued. On the 11th day of hospitalization, the results of G6PD activity and genetic testing were both normal.

Two days later, the family brought self-purchased pyrimethamine and sulfadiazine to the hospital. Following a multidisciplinary hospital-wide discussion, thorough communication of the medication risks with the family, and the signing of informed consent, standard treatment was initiated according to the regimen formulated by the clinical pharmacist: oral pyrimethamine (2 mg/kg once daily for 2 days, followed by 1 mg/kg once daily for 6 months, then adjusted to three times per week), oral sulfadiazine (50 mg/kg twice daily), and oral folinic acid (10 mg three times per week). During treatment, the dosage was adjusted according to the infant’s body weight, and close monitoring of jaundice, complete blood count, and liver and kidney function was performed. The infant tolerated the treatment well, with no significant drug-related adverse reactions during hospitalization. Jaundice resolved, and complete blood count (Figure 3) as well as liver and kidney function tests remained within normal limits. The infant was discharged after improvement.

**Figure 3:**
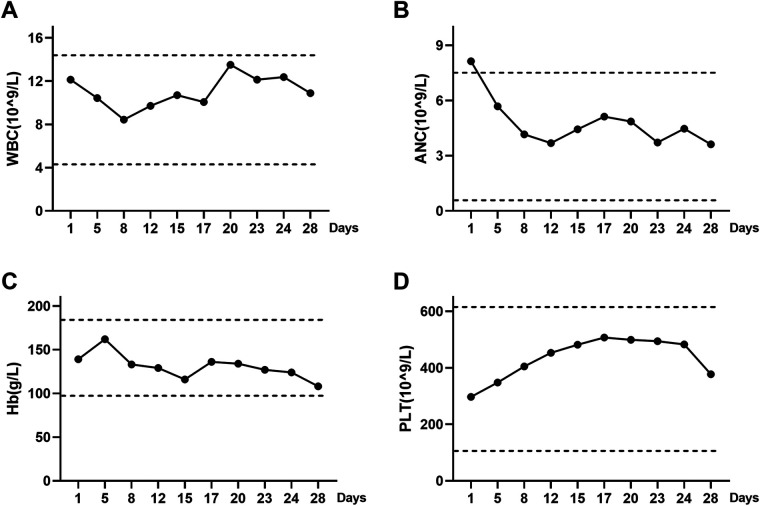
Complete blood count during hospitalization. **(A)** White blood cell count (WBC), reference range 4.3–14.2 × 10⁹/L; **(B)** Absolute neutrophil count (ANC), reference range 0.6-7.5 × 10⁹/L; **(C)** Hemoglobin (Hb), reference range 97-183 g/L; **(D)** Platelet count (PLT), reference range 100-614 × 10⁹/L.

The infant continued the above regimen after discharge and was followed up regularly in the outpatient clinic for one year. At 3 months and 7 months after discharge, repeat testing for *Toxoplasma* IgM antibody was negative. Follow-up MRI revealed only slightly widened bilateral temporal extracerebral spaces, with no other significant abnormalities on brain MRI (Figure 4), and ophthalmologic examination was also normal (Figure 2B). During the follow-up period, monitoring of complete blood count, liver and kidney function, and growth and development assessments all showed no significant abnormalities. Over the course of one year of treatment and follow-up, the family expressed satisfaction with the clinical outcome and regarded the full communication and shared decision-making as key factors that supported their adherence to the treatment protocol. A detailed timeline of the patient’s clinical course and treatment is depicted in Figure 5.

**Figure 4:**
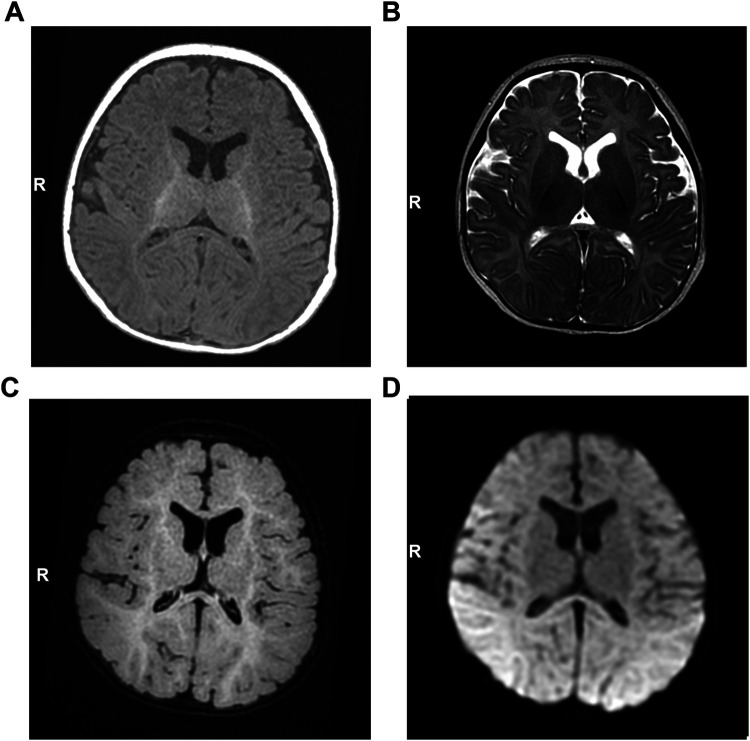
Follow-up cranial MRI at 4 months after anti-*Toxoplasma* therapy. **(A)**, **(B)**, **(C)**, and **(D)** represent different views of the same examination, demonstrating closure of the cavum septum pellucidum, consistent with normal development.

**Figure 5:**
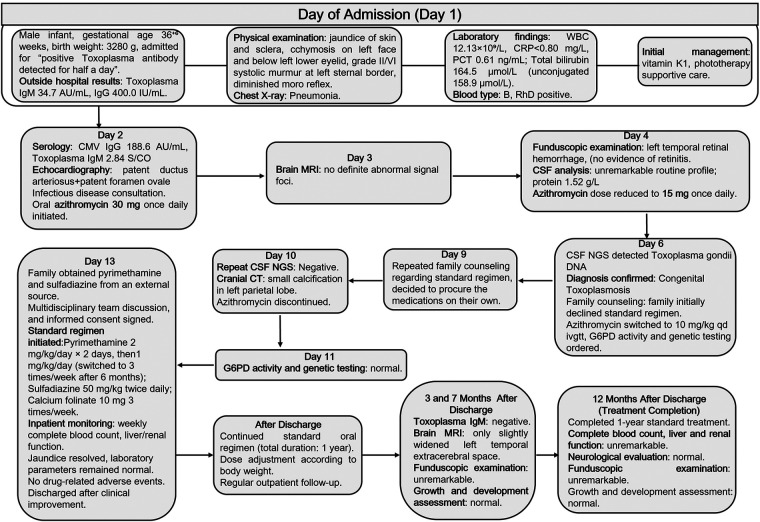
Timeline of diagnosis, treatment, and follow-up of the case.

## 3 Discussion

### 3.1 Epidemiology and pathogenesis

Congenital toxoplasmosis is caused by *T. gondii*, an intracellular protozoan parasite (8). Congenital infection typically occurs when the mother experiences a primary infection during pregnancy, after which tachyzoites are transmitted vertically across the placenta to the fetus (9). *T. gondii* completes its sexual reproductive cycle exclusively in felids. Oocysts are shed in cat feces and require sporulation under suitable environmental conditions for 1–5 days before becoming infectious (10). In this case, the family kept a cat at home, and maternal infection through contact with cat feces containing T gondii oocysts cannot be ruled out. However, other transmission routes, including ingestion of undercooked meat containing tissue cysts and consumption of vegetables or fruit contaminated with oocysts, are equally plausible. As serological testing of the cat was not performed, the cat could not be definitively identified as the source of infection. The severity of fetal infection is closely related to gestational age at the time of maternal infection, the later the gestational age, the higher the risk of transmission, but the milder the clinical manifestations tend to be (11). Conversely, without treatment, infection during early pregnancy often leads to fetal death or severe neurological and ophthalmological sequelae (12). Given that the infant’s mother did not receive anti-*Toxoplasma* therapy during pregnancy and that the infant presented with a subclinical form of the disease, it is presumed that infection likely occurred in the late stage of pregnancy. Additionally, factors such as parasite strain virulence, parasitic burden, and host immune response may also influence the severity of congenital toxoplasmosis (13–15).

### 3.2 Diagnostic approach and clinical classification

Diagnosis in neonates relies on the detection of *Toxoplasma*-specific IgM or IgA antibodies, or on molecular detection of *T. gondii* DNA in CSF (16, 17). In this case, the infant was found to have positive *Toxoplasma* IgM and IgG antibodies shortly after birth. For infants with positive serology but no clinical symptoms, confirmatory diagnosis requires repeat serological testing and CSF analysis (18, 19). In this infant, repeat CSF NGS detected *T. gondii* DNA, thereby confirming the diagnosis. The internationally accepted gold standard for the diagnosis of congenital toxoplasmosis is the persistence of anti-*Toxoplasma* IgG beyond 12 months of age or the detection of *T. gondii* DNA in body fluids or tissues, the latter traditionally accomplished by PCR (20, 21). In clinical practice, however, the sensitivity of CSF PCR is limited, and the risk of false-negative results is considerable, particularly when the parasite burden is low or empirical anti-*Toxoplasma* therapy has already been initiated (3, 22). In the present case, empirical azithromycin had been started before the CSF sample for NGS was obtained, and the parasite load may therefore have been low. Metagenomic next-generation sequencing (mNGS), an unbiased broad-spectrum pathogen detection technology, has been recommended in China as a supplementary diagnostic tool for infections caused by rare or unexpected pathogens (23). Recent case reports have also demonstrated that mNGS can successfully detect *T. gondii* DNA in seronegative immunocompromised patients, thereby overcoming some of the limitations of conventional methods (24). In our patient, serum *Toxoplasma* IgM and IgG were unequivocally positive, and CSF NGS further identified *T. gondii* DNA. The combination of serological and molecular evidence thus provides a reliable basis for the diagnosis of congenital toxoplasmosis.

The serological findings should be interpreted in the context of the kinetic characteristics of anti-*Toxoplasma* antibodies in neonates. IgG antibodies cross the placenta; therefore, a positive IgG result alone requires differentiation between passive maternal transfer and congenital infection. In this infant, however, the IgG titer was markedly elevated (400.0 IU/mL) and was accompanied by a positive IgM result. Because IgM does not cross the placenta, the detection of *Toxoplasma*-specific IgM in neonatal serum is highly suggestive of congenital infection (25). Repeated testing during the first year of follow-up showed persistently negative IgM results, further corroborating the diagnosis. As passively transferred maternal IgG is generally cleared within 12 months after birth (26), IgG testing was not repeated. Although *Toxoplasma* IgA was not measured in this case, IgA likewise does not cross the placenta and its detection in combination with IgM can improve diagnostic sensitivity. In the present case, the concurrent positivity of IgM and CSF NGS is sufficient to establish the diagnosis, and the absence of IgA testing does not compromise the reliability of the diagnostic conclusion.

On hospital day 2, CMV IgG was detected at 188.6 AU/mL. However, CMV IgM was not detected. Isolated CMV IgG positivity in a neonate typically reflects passive transfer of maternal antibodies and does not constitute evidence of congenital CMV infection. Furthermore, the infant exhibited none of the common clinical manifestations of congenital CMV infection—such as hepatosplenomegaly, thrombocytopenia, microcephaly, or sensorineural hearing loss—and CSF NGS detected only *T. gondii* DNA without any CMV-specific sequences. The intracranial calcification and elevated CSF protein in this case are therefore adequately explained by congenital toxoplasmosis.

Furthermore, when congenital infection is highly suspected, CSF analysis, ophthalmologic evaluation, and neuroimaging are recommended to identify abnormalities such as elevated CSF protein, intracranial calcifications, or retinal scars (27–30). The most characteristic imaging findings of *Toxoplasma* encephalopathy include multiple hypodense or ring-enhancing lesions in the basal ganglia and at the gray-white matter junctions, often accompanied by edema and mass effect. In this case, cranial magnetic resonance imaging revealed no definite abnormal signal foci, ophthalmologic examination identified a hemorrhagic spot in the left temporal region, and CSF protein was elevated (1.52 g/L). The infant remained free of fever, seizures, feeding difficulties, and other systemic symptoms throughout the hospital stay. Objective investigations, however, revealed evidence of mild central nervous system involvement—CSF protein was markedly elevated at 1.52 g/L, exceeding the upper reference limit of 1.0 g/L for neonates, and cranial CT demonstrated a small calcification in the left parietal lobe. Funduscopic examination detected a retinal hemorrhage in the left temporal region. It is noteworthy, however, that the initial ophthalmologic evaluation revealed none of the characteristic features of active toxoplasmic chorioretinitis, such as well-circumscribed yellowish-white retinal infiltrates with overlying vitritis, and by the end of the follow-up period no typical pigmented chorioretinal scars indicative of healed toxoplasmic chorioretinitis were observed (31). Consequently, the retinal hemorrhage was not regarded as evidence of active ocular toxoplasmosis in the clinical classification of this case. According to the classification system of the European Multicenter Study on Congenital Toxoplasmosis (EMSCOT) (21), the infant was classified as having “mildly symptomatic congenital toxoplasmosis”.

### 3.3 Treatment and management

On the basis of the standard classification, this case is designated as “mildly symptomatic congenital toxoplasmosis”—that is, the infant had no systemic clinical symptoms but showed objective evidence of subclinical mild central nervous system involvement. Within the EMSCOT classification framework, this category lies at the milder end of the clinical spectrum together with “asymptomatic” infection. However, its management principles and treatment recommendations are identical to those for symptomatic cases. Standard anti-*Toxoplasma* therapy should be initiated as soon as possible to prevent long-term sequelae, in particular the development or progression of chorioretinitis and neurodevelopmental impairment (27). Indeed, compared with truly asymptomatic infection, infants with subclinical neurological involvement may derive even greater benefit from standard treatment in terms of long-term prognosis (5). For mildly symptomatic congenital toxoplasmosis, the goal of early antiparasitic treatment is to suppress tachyzoite activity and prevent late sequelae such as neurodevelopmental disorders and retinal disease (29, 32).Notably, even in infants born with severe central nervous system involvement such as microcephaly or hydrocephalus, most can achieve favorable overall prognosis and neurodevelopmental outcomes after standardized treatment (33). Therefore, all diagnosed neonates should initiate treatment as soon as possible.

Currently available anti-*Toxoplasma* drugs include pyrimethamine, sulfadiazine, trimethoprim, azithromycin, clindamycin, spiramycin, clarithromycin, atovaquone, and dapsone, among others (6). When congenital toxoplasmosis was highly suspected in this infant, azithromycin was chosen as the initial therapy due to the lack of routine access to the standard drugs pyrimethamine and sulfadiazine in China, as well as considerations of drug availability and safety. Azithromycin has been shown to inhibit *T. gondii* tachyzoites *in vitro* by suppressing protein synthesis (34). Additionally, azithromycin achieves high concentrations in tissues and cells, demonstrating activity against the parasite *in vivo* (35). To date, only a single center in China has reported a case of azithromycin use in neonatal congenital toxoplasmosis (7). This report represents a single-center experience and lacks systematic efficacy evaluation and long-term follow-up data. The definitive efficacy of azithromycin in congenital toxoplasmosis therefore remains to be established. International reviews explicitly state that the efficacy of azithromycin monotherapy for toxoplasmosis remains unclear, and available clinical evidence primarily supports its use in combination with pyrimethamine (4). In this case, therefore, the administration of azithromycin essentially represented empirical bridging therapy under conditions of limited drug accessibility rather than an alternative to the standard regimen. In the treatment of ocular toxoplasmosis, the efficacy of azithromycin combined with pyrimethamine or trimethoprim-sulfamethoxazole is comparable to that of the standard regimen, with a lower incidence of adverse reactions (36–38). After azithromycin therapy, repeat CSF NGS for *T. gondii* turned negative. However, this result cannot be independently attributed to the therapeutic effect of azithromycin, as the reduction in CSF parasite burden may also have been influenced by confounding factors such as host immune clearance. NGS is currently employed primarily for pathogen diagnosis rather than therapeutic monitoring. Azithromycin is associated with potential adverse effects including elevated liver enzymes and QT interval prolongation, necessitating daily electrocardiographic monitoring and periodic liver function testing.

Although treatment protocols have not been standardized and clinical practices vary considerably across the globe, the preferred regimen remains the combination of pyrimethamine and sulfadiazine, supplemented with folinic acid (3, 4, 6). The efficacy of this regimen has been validated in several clinical studies. The National Collaborative Chicago-Based Congenital Toxoplasmosis Study prospectively followed 120 infants with congenital toxoplasmosis and demonstrated that all infants without substantial neurologic disease at birth achieved normal cognitive, neurologic, and auditory outcomes after 1 year of treatment, while more than 72% of those with moderate or severe neurologic disease at birth also attained normal outcomes (5). This high-quality long-term follow-up evidence provides the core evidence base for recommending this regimen as first-line therapy. This drug combination synergistically inhibits dihydropteroate synthase (DHPS) and dihydrofolate reductase (DHFR), two key enzymes in folate biosynthesis, which is essential for the growth and replication of *T. gondii* (39). Moreover, the clinical and preclinical data supporting the use of pyrimethamine-sulfadiazine are the most comprehensive, particularly in immunocompromised patients (40). All studies have demonstrated that this combination exhibits high activity and good penetration into tissues such as the brain (41). Therefore, the pyrimethamine-sulfadiazine combination was selected as the first-line choice. In this case, through multidisciplinary collaboration and thorough communication with the family, after obtaining informed consent from the parents who sourced the medications themselves and after excluding G6PD deficiency, the standard treatment was implemented strictly according to an individualized dosing regimen.

### 3.4 Safety monitoring

The major risks associated with this regimen are related to hematologic system involvement and allergic reactions. Pyrimethamine can cause dose-related bone marrow suppression, primarily manifesting as neutropenia, and increasing the dose of folinic acid usually helps to alleviate this condition (42). Sulfadiazine may cause allergic reactions such as rash (43) and, in rare cases, can lead to severe neutropenia on its own (44). When used in combination, the incidence of hematologic adverse events in neonates can be as high as 30%, with a higher frequency during the first two months of life (28). Therefore, concomitant use of other sulfonamides (such as trimethoprim-sulfamethoxazole) must be strictly avoided during treatment to prevent the induction of severe or persistent bone marrow suppression (33). Given these risks, close monitoring is required during therapy. After initiating daily pyrimethamine dosing, a complete blood count should be monitored weekly. If values remain stable, the monitoring interval may be extended to every two weeks, and then to monthly. When the ANC falls below 0.8 × 10⁹/L, treatment should be temporarily interrupted and resumed after recovery (28). Liver and kidney function should be assessed every three to six months (28). Throughout the 12-month treatment and follow-up period, the infant tolerated the standard regimen well. Hematologic monitoring showed that the ANC remained consistently above 1.2 × 10⁹/L and never reached the threshold for treatment suspension (ANC < 0.8 × 10⁹/L) (28). Hemoglobin remained stable within the range of 92–125 g/L, consistent with physiologic anemia of infancy, and neither blood transfusion nor pharmacologic intervention was required. The platelet count was consistently above 150 × 10⁹/L, and no bleeding events occurred. With respect to hepatic function, both ALT and AST remained below 1.5 times the upper limit of normal, and serum creatinine levels were normal throughout. No sulfonamide-related adverse reactions—such as rash, fever, or gastrointestinal intolerance—were reported, and no dose suspension or adjustment due to adverse events was necessary. These longitudinal monitoring data confirm the safety of this regimen in a Chinese neonate under rigorous surveillance.

### 3.5 Follow-Up and prevention

Given that *Toxoplasma* infection can lead to late-onset sequelae, long-term clinical follow-up throughout early childhood is recommended even for mildly symptomatic infants. Follow-up should include serological testing, ophthalmologic examination, hearing assessment, neurological evaluation, and neurodevelopmental monitoring (33, 45). The infant was followed up for one year after discharge, during which *Toxoplasma* IgM antibodies remained persistently negative, and complete blood count, liver and kidney function, and magnetic resonance imaging all remained unremarkable, with growth and development assessments falling within normal ranges. It is important to emphasize that the absence of abnormalities on imaging does not necessarily indicate that the central nervous system is unaffected, particularly in the early stages of infection or when involvement is mild. The lack of new lesions on follow-up imaging during treatment suggests that the therapy was effective and that the disease was well controlled, with no evidence of progression in central nervous system involvement. However, chorioretinitis associated with congenital toxoplasmosis may first appear or recur years or even decades after the completion of treatment, and the 1-year follow-up period is therefore insufficient to exclude late ophthalmologic or neurological sequelae. Continued ophthalmologic and neurodevelopmental surveillance throughout childhood is warranted for this infant.

The cornerstone of congenital toxoplasmosis prevention lies in preconception and prenatal management. For seronegative women of childbearing age, *Toxoplasma* serological screening should be performed before conception or during early pregnancy to establish baseline immune status. Those who test seronegative should receive health education on hygiene measures during pregnancy, including avoidance of undercooked meat, thorough handwashing after contact with cat feces, wearing gloves when handling soil, and washing fruits and vegetables thoroughly before consumption (46). In countries and regions with well-established prenatal care systems, periodic serological monitoring for *Toxoplasma* during pregnancy is recommended for all pregnant women or those at high risk, enabling early detection of maternal seroconversion and prompt initiation of anti-*Toxoplasma* therapy, thereby reducing the risk of vertical transmission and the severity of fetal infection (47). These preventive measures apply equally to immunocompromised individuals. In addition, timely anti-*Toxoplasma* treatment during pregnancy in women with confirmed maternal infection is an effective measure for preventing fetal infection (4).

### 3.6 Limitations

This report has several limitations. The CSF NGS testing was performed by a third-party clinical laboratory. Owing to the information gap between the clinical requesting party and the third-party laboratory, we were unable to obtain the full technical parameters, including the library preparation kit, bioinformatics pipeline, and internal quality control data. The *T. gondii* DNA detected in CSF by NGS was not independently confirmed by an orthogonal method (qPCR), which limits the strength of the molecular confirmation. *Toxoplasma* IgA testing was not performed, and serial IgG titers were not tracked longitudinally, resulting in an incomplete serological assessment. The conversion of CSF NGS to negative following azithromycin therapy cannot be independently attributed to the therapeutic effect of azithromycin, as the reduction in CSF parasite burden may have been confounded by host immune clearance and the timing of lumbar puncture. Furthermore, as systematic *Toxoplasma* serological screening and fetal diagnostic investigations were not performed during the mother’s pregnancy, her infection status, the precise timing of maternal infection, and the presence of fetal indicators of infection remain unknown. This limits our ability to precisely infer the timing of vertical transmission. Finally, chorioretinitis associated with congenital toxoplasmosis may first appear or recur years or even decades after the completion of treatment (4). The 1-year follow-up in this case is therefore insufficient to assess long-term ocular outcomes and continued ophthalmologic and neurodevelopmental surveillance is warranted.

## Conclusion

This work reports a case of mildly symptomatic congenital toxoplasmosis in a neonate diagnosed by CSF NGS, in which the standard treatment regimen of pyrimethamine combined with sulfadiazine was successfully implemented under conditions of limited drug accessibility in China. The infant was found to have positive *Toxoplasma* IgG and IgM antibodies through routine screening after birth, and the diagnosis was subsequently confirmed through systematic evaluation. In the initial phase of treatment, due to the lack of routine access to standard drugs in China, the situation posed a dilemma of having a diagnosis without available therapeutic agents; therefore, empirical therapy with azithromycin was administered. Ultimately, through repeated discussions and comprehensive communication with the family, and after obtaining informed consent from the parents who sourced the medications themselves, combined therapy with pyrimethamine, sulfadiazine, and folinic acid was initiated according to an individualized pharmaceutical regimen. The infant tolerated the treatment well throughout the course, with no serious adverse reactions, and follow-up indicated a favorable early prognosis.

This case demonstrates that, despite significant barriers to drug accessibility, the successful application of an internationally standardized regimen for congenital toxoplasmosis is feasible in China through standardized shared decision-making with the family, rigorous treatment monitoring, and support from a multidisciplinary team. This case provides a referenceable approach for addressing similar therapeutic challenges in rare diseases. We suggest that future efforts consider establishing regional emergency supply mechanisms for rare disease therapeutic drugs and developing diagnostic and treatment consensus guidelines tailored to national conditions, with the aim of systematically improving the diagnosis, treatment, and long-term prognosis of children with congenital toxoplasmosis.

## Data Availability

The raw data supporting the conclusions of this article will be made available by the authors, without undue reservation.

## References

[1] Walana W Odai SA Tamomh AG. Prevalence, risk factors, diagnosis and outcomes of toxoplasma gondii infection in pregnancy: a review. Parasitol Int. (2026) 110:103143. 10.1016/j.parint.2025.103143 40818495

[2] Gantt S Binquet C Lejeune C Seror V Peyron F Bertaux A-C. The cost-effectiveness of neonatal versus prenatal screening for congenital toxoplasmosis. PLoS One. (2019) 14(9):e0221709. 31532766 10.1371/journal.pone.0221709 PMC6750576

[3] Elsheikha HM Marra CM Zhu X-Q. Epidemiology, pathophysiology, diagnosis, and management of cerebral toxoplasmosis. Clin Microbiol Rev. (2020) 34(1):e00115–19. 10.1128/CMR.00115-19 33239310 PMC7690944

[4] Ildiko Rita Dunay KG Dhakal R Liesenfeld O Montoya JG. Treatment of toxoplasmosis: historical perspective, animal models, and current clinical practice. Clin Microbiol Rev. (2018) 31(4):e00057–17. 10.1128/CMR.00057-17 30209035 PMC6148195

[5] McLeod R Boyer K Karrison T Kasza K Swisher C Roizen N. Outcome of treatment for congenital toxoplasmosis, 1981–2004: the national collaborative ChicagoBased, congenital toxoplasmosis study. Clin Infect Dis. (2006) 42(10):1383–94. 10.1086/501360 16619149

[6] Aerts R Mehra V Groll AH Martino R Lagrou K Robin C. Guidelines for the management of toxoplasma gondii infection and disease in patients with haematological malignancies and after haematopoietic stem-cell transplantation. Guidelines from the 9th European Conference on Infections in Leukaemia, 2022. Lancet Infect Dis. (2024) 24(5):e291–306. 10.1016/S1473-3099(23)00495-4 38134949

[7] Li J Zhao J Yang X Wen Y Huang L Ma D. One severe case of congenital toxoplasmosis in China with good response to azithromycin. BMC Infect Dis. (2021) 21(1):920. 10.1186/s12879-021-06619-1 34488656 PMC8422759

[8] Teixeira SC de Souza G dos Santos NCL de Oliveira RM Silva NBS de Lima Junior JP. Brazilian Red propolis and its active constituent 7-O-methylvestitol impair early and late stages of toxoplasma gondii infection in human placental models. Microorganisms. (2025) 13(8):1937. 10.3390/microorganisms13081937 40871442 PMC12388039

[9] de Oliveira RM Lima dos Santos NC Almeida MPO Lima Júnior JPD Souza GD Barbosa MC. Novel drug candidates targeting toxoplasma gondii in maternal–fetal interface models. Front Pharmacol. (2025) 16:1673462. 10.3389/fphar.2025.1673462 41415569 PMC12708611

[10] He M Zhang B Han S Qian J He Z Wei Y. Development and optimization of an ELISA method to detect toxoplasma gondii oocyst infection in cats. Parasitol Res. (2025) 124(7):79. 10.1007/s00436-025-08523-y 40619535 PMC12229958

[11] Group TSSRoCTs. Effectiveness of prenatal treatment for congenital toxoplasmosis: a meta-analysis of individual patients’ data. Lancet. (2007) 369(9556):115–22. 10.1016/S0140-6736(07)60072-5 17223474

[12] Garozzo MT Garozzo R Betta P Cilauro S Saporito A D'Amico P. Congenital toxoplasmosis: an observational retrospective study in the eastern sicily. Front Pediatr. (2025) 13:1597001. 10.3389/fped.2025.1597001 40656197 PMC12245800

[13] Rico-Torres CP Vargas-Villavicencio JA Correa D. Is toxoplasma gondii type related to clinical outcome in human congenital infection? Systematic and critical review. Eur J Clin Microbiol Infect Dis. (2016) 35(7):1079–88. 10.1007/s10096-016-2656-2 27146878

[14] Toland AE Jamieson SE de Roubaix L-A Cortina-Borja M Tan HK Mui EJ. Genetic and epigenetic factors at COL2A1 and ABCA4 influence clinical outcome in congenital toxoplasmosis. PLoS One. (2008) 3(6):e2285. 18523590 10.1371/journal.pone.0002285 PMC2390765

[15] Campos MAS Gilbert RE Freeman K Lago EG Bahia-Oliveira LMG Tan HK. Ocular sequelae of congenital toxoplasmosis in Brazil compared with Europe. PLoS Negl Trop Dis. (2008) 2(8):e277. 18698419 10.1371/journal.pntd.0000277 PMC2493041

[16] Pinon JM Foudrinier F Mougeot G Cathy Marx DA Toupance O Niel G. Evaluation of risk and diagnostic value of quantitative assays for anti-toxoplasma gondii immunoglobulin A (IgA), IgE, and IgM and analytical study of specific IgG in immunodeficient patients. J Clin Microbiol. (1995) 33(4):878–84. 10.1128/jcm.33.4.878-884.1995 7790453 PMC228059

[17] Wallon M Caudie C Rubio S Bellini L Girault V Gay-Andrieu F. Value of cerebrospinal fluid cytochemical examination for the diagnosis of congenitaltoxoplasmosis at birth in France. Pediatr Infect Dis J. (1998) 17(8):705–10. 10.1097/00006454-199808000-00009 9726345

[18] Suryanarayana AU Balasubramaniam VV Prashanthi K Gochhait D Pari H. Detection of toxoplasma gondii tachyzoites in cerebrospinal fluid: once in a blue moon finding. Cytopathology. (2025) 36(6):629–31. 10.1111/cyt.70005 40548643

[19] Vera CN Linam WM Gadde JA Wolf DS Walson K Montoya JG. Congenital toxoplasmosis presenting as eosinophilic encephalomyelitis with spinal cord hemorrhage. Pediatrics. (2020) 145(2):e20191425. 10.1542/peds.2019-1425 31941759

[20] Machado AS Andrade GM Januário JN Fernandes MD Carneiro ACA Carneiro M. Igg and IgM western blot assay for diagnosis of congenital toxoplasmosis. Mem Inst Oswaldo Cruz. (2010) 105(6):757–61. 10.1590/S0074-02762010000600005 20944989

[21] Lebech M Joynson DHM Seitz HM Thulliez R Gilbert RE Dutton GN. Classification system and case definitions of toxoplasma gondii infection in immunocompetent pregnant women and their congenitally infected offspring. Eur J Clin Microbiol Infect Dis. (1996) 15(10):799–805. 10.1007/BF01701522 8950557

[22] Anselmo LMP Vilar FC Lima JE Yamamoto AY Bollela VR Takayanagui OM. Usefulness and limitations of polymerase chain reaction in the etiologic diagnosis of neurotoxoplasmosis in immunocompromised patients. J Neurol Sci. (2014) 346(1-2):231–4. 10.1016/j.jns.2014.08.034 25240445

[23] Cytology CSoIDaCF. Expert consensus on clinical application of metagenomic next-generation sequencing of cerebrospinal fluid in the diagnosis of infectious diseases of the central nervous system. Chin J Neurol. (2021) 54(12):1234–40.

[24] Lv Q Cao Y Wang J Meng L Wang G. Metagenomic next-generation sequencing may be a tool for timely diagnosis of seronegative and primary toxoplasma infection after allogeneic hematopoietic stem cell transplantation: a. Case Report and Literature Review. Pediatr Infect Dis J. (2023) 43(2):155–9. 10.1097/INF.0000000000004170 38190489

[25] Arkhis S Rouges C Dahane N Guegan H Yéra H Robert-Gangneux F. Could PLATELIA toxo IgM be the new gold standard for the serological diagnosis of congenital toxoplasmosis: a French multicenter study. J Clin Microbiol. (2024) 62(2):e0122223. 10.1128/jcm.01222-23 38259072 PMC10865801

[26] Montoya JG Liesenfeld O. Toxoplasmosis. Lancet. (2004) 363(9425):1965–76. 10.1016/S0140-6736(04)16412-X 15194258

[27] Maldonado YA Read JS. Diagnosis, treatment, and prevention of congenital toxoplasmosis in the United States. Pediatrics. (2017) 139(2):e20163860. 10.1542/peds.2016-3860 28138010

[28] Bollani L Auriti C Achille C Garofoli F De Rose DU Meroni V. Congenital toxoplasmosis: the state of the art. Front Pediatr. (2022) 10:894573. 10.3389/fped.2022.894573 35874584 PMC9301253

[29] Guerina NG Hsu HW Meissner HC Maguire JH Lynfield R Stechenberg B. Neonatal serologic screening and early treatment for congenital toxoplasma gondii infection. The new England regional toxoplasma working group. N Engl J Med. (1994) 330(26):1858–63. 10.1056/NEJM199406303302604 7818637

[30] de-la-Torre A Ríos-Cadavid AC Cardozo-García CM Padilla L Gómez-Marín JE. Serum levels of dehydroepiandrosterone sulfate (DHEAS) in ocular toxoplasmosis. J Microbiol Immunol Infect. (2012) 45(1):65–8. 10.1016/j.jmii.2011.09.003 22153766

[31] Park Y-H Han J-H Nam H-W. Clinical features of ocular toxoplasmosis in Korean patients. Korean J Parasitol. (2011) 49(2):167. 10.3347/kjp.2011.49.2.167 21738273 PMC3121074

[32] Chen N Cai Q Wang S Song Q Xie Y Shi H. Evaluation of the efficicacy of myrcene in the treatment of eimeria tenella and toxoplasma gondii infection. J Vet Med Sci. (2025) 87(1):32–42. 10.1292/jvms.24-0397 39567006 PMC11735216

[33] McAuley J Boyer KM Patel D Mets M Swisher C Roizen N. Early and longitudinal evaluations of treated infants and children and untreated historical patients with congenital toxoplasmosis: the Chicago collaborative treatment trial. Clin Infect Dis. (1994) 18(1):38–72. 10.1093/clinids/18.1.38 8054436

[34] Abugri DA Wijerathne SVT Sharma HN Ayariga JA Napier A Robertson BK. Quercetin inhibits toxoplasma gondii tachyzoite proliferation and acts synergically with azithromycin. Parasit Vectors. (2023) 16(1):261. 10.1186/s13071-023-05849-3 37537675 PMC10401810

[35] Lopes CD Silva NM Ferro EAV Sousa RA Firmino ML Bernardes ES. Azithromycin reduces ocular infection during congenital transmission of toxoplasmosis in the calomys callosus model. J Parasitol. (2009) 95(4):1005–10. 10.1645/GE-1765.1 20050006

[36] Balaskas K Vaudaux J Boillat-Blanco N Guex-Crosier Y. Azithromycin versus sulfadiazine and pyrimethamine for non-vision-threatening toxoplasmic retinochoroiditis: a pilot study. Med Sci Monit. (2012) 18(5):CR296–302. 10.12659/MSM.882735 22534709 PMC3560626

[37] Rothova A Bosch-Driessen LE van Loon NH Treffers WF. Azithromycin for ocular toxoplasmosis. Br J Ophthalmol. (1998) 82(11):1306–8. 10.1136/bjo.82.11.1306 9924338 PMC1722414

[38] Lashay A Mirshahi A Parandin N Riazi Esfahani H Mazloumi M Reza Lashay M. A prospective randomized trial of azithromycin versus trimethoprim/sulfamethoxazole in treatment of toxoplasmic retinochoroiditis. J Curr Ophthalmol. (2017) 29(2):120–5. 10.1016/j.joco.2016.10.002 28626822 PMC5463001

[39] Derouinl F Chastang C. *In vitro* effects of folate inhibitors on toxoplasma gondii. Antimicrob Agents Chemother. (1989) 33(10):1753–9. 10.1128/AAC.33.10.1753 2531568 PMC172750

[40] Chêne G Thiébaut R. Options for clinical trials of pre and post-natal treatments for congenital toxoplasmosis. Mem Inst Oswaldo Cruz. (2009) 104(2):299–304. 10.1590/S0074-02762009000200025 19430657

[41] Avindra Nath M Anthony P Sinai P. Cerebral toxoplasmosis. Curr Treat Options Neurol. (2003) 5(1):3–12. 10.1007/s11940-003-0018-8 12521559

[42] Carellos EVM de Andrade JQ Romanelli RMC Tibúrcio JD Januário JN Vasconcelos-Santos DV. High frequency of bone marrow depression during congenital toxoplasmosis therapy in a cohort of children identified by neonatal screening in minas gerais, Brazil. Pediatr Infect Dis J. (2017) 36(12):1169–76. 10.1097/INF.0000000000001561 28151845

[43] Asyraf PA Kusnadi IF Stefanus J Khairinisa MA Abdulah R. Clinical manifestations and genetic influences in sulfonamide-induced hypersensitivity. Drug Healthc Patient Saf. (2022) 14:113–24. 10.2147/DHPS.S347522 35903308 PMC9315057

[44] Galanakis E Manoura A Antoniou M Sifakis S Korakaki E Hatzidaki E. Outcome of toxoplasmosis acquired during pregnancy following treatment in both pregnancy and early infancy. Fetal Diagn Ther. (2007) 22(6):444–8. 10.1159/000106352 17652934

[45] Wallon M Cozon G Ecochard R Lewin P Peyron F. Serological rebound in congenital toxoplasmosis: long-term follow-up of 133 children. Eur J Pediatr. (2001) 160(9):534–40. 10.1007/s004310100805 11585075

[46] Wehbe K Pencole L Lhuaire M Sibiude J Mandelbrot L Villena I. Hygiene measures as primary prevention of toxoplasmosis during pregnancy: a systematic review. J Gynecol Obstet Hum Reprod. (2022) 51(3):102300. 10.1016/j.jogoh.2021.102300 34979320

[47] Bobić B Villena I Stillwaggon E. Prevention and mitigation of congenital toxoplasmosis. Economic costs and benefits in diverse settings. Food Waterborne Parasitol. (2019) 16:e00058. 10.1016/j.fawpar.2019.e00058 32095628 PMC7034037

